# RNAm5Cfinder: A Web-server for Predicting RNA 5-methylcytosine (m5C) Sites Based on Random Forest

**DOI:** 10.1038/s41598-018-35502-4

**Published:** 2018-11-23

**Authors:** Jianwei Li, Yan Huang, Xiaoyue Yang, Yiran Zhou, Yuan Zhou

**Affiliations:** 1 Institute of Computational Medicine, School of Artificial Intelligence, Hebei University of Technology, Tianjin, China; 2 Department of Biomedical Informatics, School of Basic Medical Sciences, Center for Noncoding RNA Medicine, Peking University, Beijing, China

## Abstract

5-methylcytosine (m5C) is a common nucleobase modification, and recent investigations have indicated its prevalence in cellular RNAs including mRNA, tRNA and rRNA. With the rapid accumulation of m5C sites data, it becomes not only feasible but also important to build an accurate model to predict m5C sites *in silico*. For this purpose, here, we developed a web-server named RNAm5Cfinder based on RNA sequence features and machine learning method to predict RNA m5C sites in eight tissue/cell types from mouse and human. We confirmed the accuracy and usefulness of RNAm5Cfinder by independent tests, and the results show that the comprehensive and cell-specific predictors could pinpoint the generic or tissue-specific m5C sites with the Area Under Curve (AUC) no less than 0.77 and 0.87, respectively. RNAm5Cfinder web-server is freely available at http://www.rnanut.net/rnam5cfinder.

## Introduction

RNA modification plays an important role in all three domains of life^1–3^. To date, more than 150 kinds of RNA modifications have been discovered, while 5-methylcytosine (m5C) is one of the most prevalent modification types^4^. Thanks to the novel applications of high-throughput sequencing technique for detecting RNA m5C modification (e.g., bisulfite sequencing and aza-IP), a pilot whole-transcriptome map of m5C sites have become available, where the modification sites mainly appear in the anticodon loop and the variable region of tRNAs and rRNAs, and the coding sequences in mRNAs^5–9^. Similar to other nucleobase modifications in RNA, m5C also influences RNA structural stability and translation efficiency, and further researches revealed that it could promote mRNA export and regulate tissue differentiation^10,11^. But the functions of m5C in RNA are still not fully understood, partly because the experimental identification of m5C sites is still expensive and labor-intensive. For this purpose, here, we developed a web-based tool named RNAm5Cfinder to predict m5C sites, which would help researchers to screen potential m5C sites easily and quickly and provide a new tool to dig functional implication of m5C.

RNAm5Cfinder is a platform with an easy-to-use web interface to predict m5C modification sites in RNA sequences. It adopts one-hot encoding for coding RNA sequences and random forest algorithm which is a supervised machine learning method for solving classification problems. In view of the fact that m5C is a tissue-specific modification, we built independent predictor for every tissue/cell type respectively. Finally, we optimized each predictor independently by cross-validation and benchmarked the predictors by independent tests. To our best knowledge, RNAm5Cfinder is the first m5C predictor that allows for predicting tissue-specific m5C sites with competitive precision.

## Results and Discussion

### Establishment of the predictor and performance benchmarking

**Figure 1:**
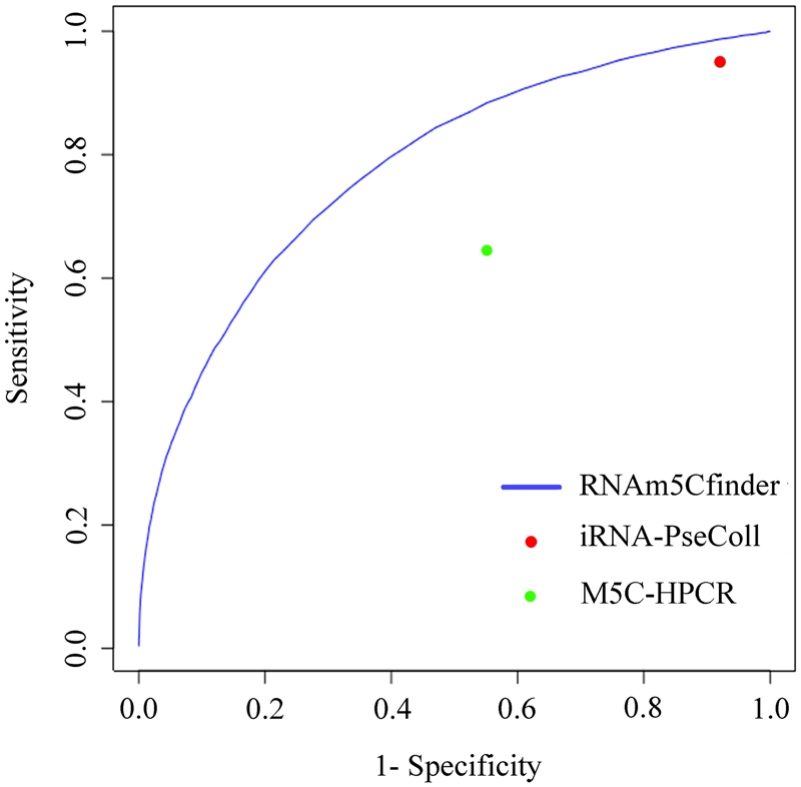
Performance comparison between RNAm5Cfinder comprehensive predictor and other available servers on independent test.

**Figure 2:**
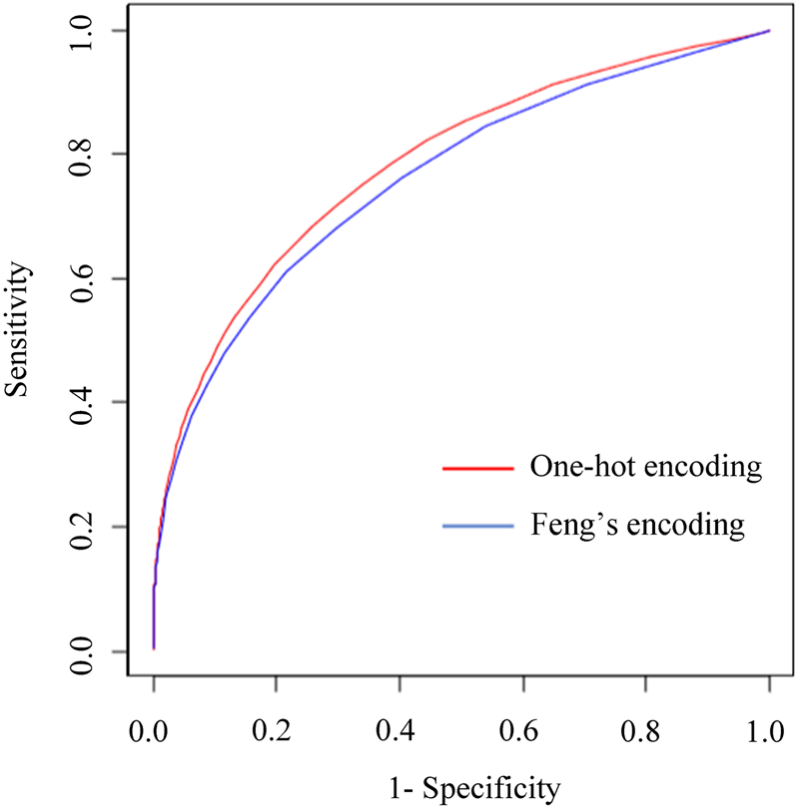
The comparison between one-hot encoding and Feng’s encoding on independent test.

The m5C modification data covering 7 tissues of mouse and human Hela cells were collected from previous studies^10,12^. We first integrated all m5C sites to build a comprehensive (generic) predictor. In the training process, we continuously optimized the ratio of the positives and the negatives of the training data set and changed the number of the decision trees in the random forest predictor by five-fold cross-validation. The results suggest that the optimal parameters are 1:30 ratio and 300 decision trees, respectively. In order to verify the performance of the predictor, we benchmarked and compared its performance with other state-of-the-art published web servers for predicting RNA m5C sites on the same independent test set. We found two available online servers for predicting RNA m5C sites which are iRNA-PseColl developed by Feng *et al*. and M5C-HPCR developed by Zhang *et al*.^13,14^. Both of them can predict m5C sites in RNA sequences, but they don’t permit tissue-specific prediction. Therefore, we compared the performance of our comprehensive predictor with iRNA-PseColl and M5C-HPCR. Note that the thresholds of the servers above are fixed, resulting in a single point in the ROC (receiver operating characteristic curve) curve that corresponds to their performance (Fig. 1). As for the strategy for coding RNA sequence, RNAm5Cfinder adopted one-hot encoding and by trying to re-train our predictor with Feng’s coding strategy and found that the performance was slightly reduced (Fig. 2), indicating that one-hot encoding is at least comparable to the current state-of-art method for RNA m5C site prediction. Another reason for picking one-hot encoding is that it is timesaving and could give the users a good experience comparing to other strategies.

### The performance of the tissue-specific predictors

**Figure 3:**
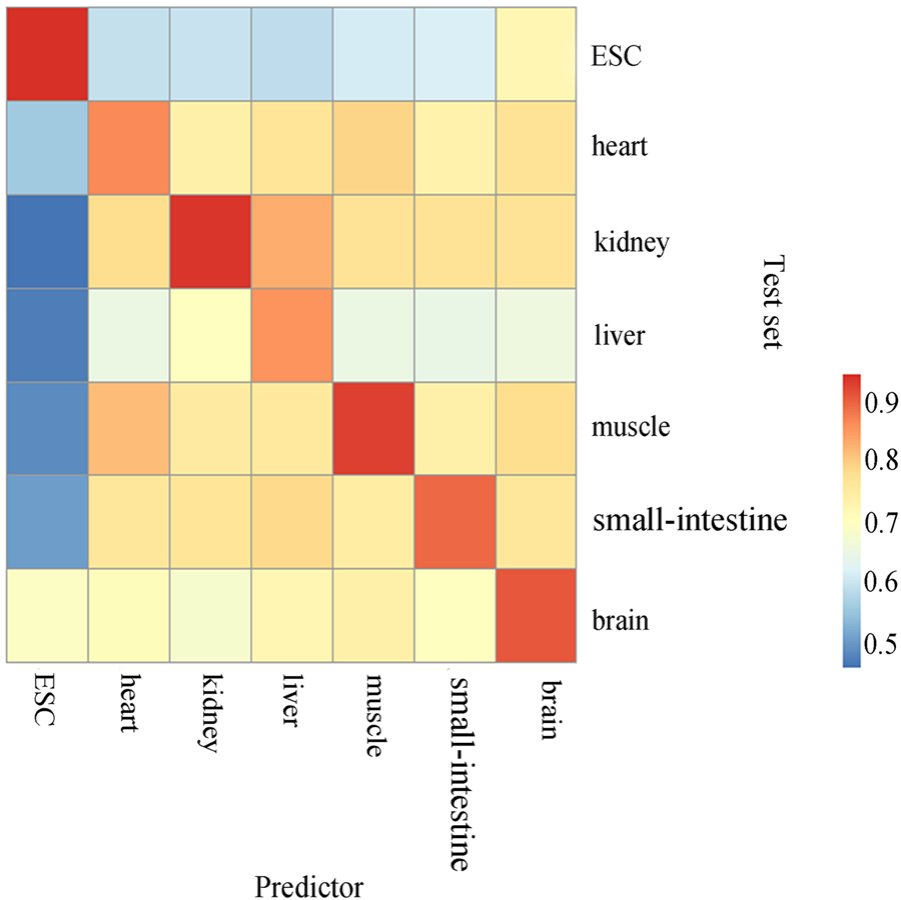
The results of intra- and inter-tissue independent tests for each tissue specific predictor. (**A**) The color correlates with the performance (AUC). (**B**) ESC, embryonic stem cell.

Taking into account the modification spectrum in different cell types or tissues are not the same, one comprehensive predictor can not accurately predict the m5C sites from each specific tissue or cell type. We further applied tissue-specific training and independent test sets where RNA m5C modification data was came from experiments on single tissue or cell to test and benchmark the tissue-specific m5C predictors (Table 1). In order to verify the robustness of the constructed tissue-specific predictors, we performed both intra- and inter-tissue independent tests for each tissue-specific predictor. For each independent test set, we removed the samples which were used to train the predictors for the rest of tissues. In other words, we only considered tissue-specific sites in the independent test for the intra- and inter-tissue independent tests. The results are summarized in Fig. 3. Clearly, the intra-tissue prediction performances, which are all above 0.87 in terms of AUC, are substantially better than inter-tissue prediction performance. This is consistent with previous studies, where m5C is implied as a tissue-specific modification^10^. This result also supports that it is necessary to build tissue-specific m5C predictors.

**Table 1:** AUC of independent test of different predictors.

Cell types	AUC
mouse_ESC	0.902
mouse_Heart	0.772
mouse_Kidney	0.768
mouse_Liver	0.768
mouse_Muscle	0.767
mouse_Small-Intestine	0.769
mouse_Brain	0.775
human_Hela	0.765

### The construction of RNAm5Cfinder web-server

To facilitate the community, we built a web-server named RNAm5Cfinder with the optimized comprehensive and tissue-specific predictors mentioned above. RNAm5Cfinder has a user-friendly interface and step-by-step guide. It takes the FASTA sequences as the input and provide the option to switch between the comprehensive predictor and the tissue-specific predictors. We also provide 3 levels of stringent thresholds, corresponding to the false positive rate values of 1%, 5%, 10%. Considering users may analyze large dataset which will spend plenty of time, RNAm5Cfinder also supports the function to send results to the submitted E-mail address.

## Methods

### Datasets

We gathered three available m5C datasets in GEO database including GSE90963 (human Hela cell), GSE93749 (human Hela cell; heart, muscle, brain, kidney and liver of mouse) and GSE83432 (mouse ESC and brain). Then m5C sites from the three datasets were first mapped to the Ensembl transcripts (queried at Feb, 2018, the genome version is GRCh37 for human and GRCm38 for mouse)^15^. For multiple transcripts of the same gene, we picked the mRNA transcript which have relatively more modification sites to insure the quality and reliability of data. One quarter of the m5C site data was randomly selected as the independent test set while the rest was used to train the predictors. The negative samples were randomly selected from the non-modified C sites in the transcripts. Since the ratio of positive and negative training samples could affect the precision of the prediction model, we preliminarily tested 3 ratios (1:10, 1:30, 1:50) and finally considered the best one (1:30) based on cross-validation. In order to fit the real-world data, as for the independent test sets, all of the non-modified C sites were used as the negative samples (Table 2).

**Table 2:** The sample size of different tissues’ training and test datasets.

Tissues	Training set		Test set	
	pos	neg	pos	neg
Comprehensive	19,798	593,941	6636	1,924,243
ESC^a^-specific	3440	103,201	828	299,610
Heart-specific	12,703	381,091	100	30,433
Kidney-specific	12,700	381,001	122	37,088
Liver-specific	11,937	358,111	125	37,844
Muscle-specific	11,826	354,781	118	36,519
Small-Intestine-specific	11,372	341,161	107	32,170
Brain-specific	19,141	424,231	472	155,409

The ratio of the positives and the negatives of the training set and test set were set to 1:30 and 1:all respectively. As for the test sets of tissue-specific predictors, samples which were used to train predictors for the other tissues were discarded.

^a^ESC, embryonic stem cell.

### Sequence encoding

To train the machine learning model, the RNA sequence flanking the modified/non-modified sites should be translated to the numeric feature encoding. In this study, two kinds of encoding strategies were tested and compared, which were the one-hot encoding^16^ and Feng’s encoding^14^. The one-hot encoding uses *n* bits of 0 or 1 to represent *n* kinds of nucleotide state. For each position, the A, G, C, T are translated into vectors of (1, 0, 0, 0), (0, 1, 0, 0), (0, 0, 1, 0) and (0, 0, 0, 1), respectively. Feng’s encoding also uses four bits to represent specific nucleotide. But unlike one-hot encoding, the first three bits in Feng’s encoding represent three kinds of physicochemical characters (which are the ring number, the chemical functionality and the number of hydrogen bonds). And the fourth bit of Feng’s encoding represents the accumulated occurrence frequency of the nucleotide in the sequence. Therefore, A, G, C, T are translated into vectors of (1, 1, 1, FreqA), (1, 0, 0, FreqG), (0, 1, 0, FreqC) and (0, 0, 1, FreqT), respectively. The size of flanking sequence window to be encoded by the one-hot and Feng’s encodings are both 10, which were optimized by five-fold cross-validation. According to their performance and complexity we finally chose one-hot encoding strategy.

### Machine learning algorithm

We have tested four methods of machine learning which are logistic regression, naïve Bayes, Decision Tree (with parameters minsplit = 35, cp = 0.00001 and maxdepth = 30) and Random forest (RF) with integrated RNA m5C sites. The performance of each algorithm is shown in Table 3. Considering both efficiency and accuracy, we finally chose RF as our preferred algorithm. RF algorithm is a robust machine learning framework that has been widely used in medicine and biology information fields^17^. RF consists of a large ensemble of classification and regression trees (CARTs). The number of CARTs is defined as n_tree, which was also optimized by cross-validation. The random forest algorithm was implemented by using the ‘randomForest’ package in R^18^.

**Table 3:** Performance of different machine learning algorithm.

Algorithm	AUC
logistic regression	0.700
naïve Bayes	0.686
Decision Tree	0.726
Random forest	0.773

### Performance evaluation

In this study we used ROC (receiver operating characteristic) curve, which is less affected by the unbalanced test data set, to evaluate the performance of predictors. ROC curve reflects the overall relationship between sensitivity and specificity when different thresholds are applied. The sensitivity and specificity are defined as

1 $$Sensitivity=\frac{TP}{TP+FN}$$

2 $$Specificity=\frac{TN}{TN+FP}$$

where TP, TN, FP and FN represent the number of true positive, true negative, false positive and false negative samples, respectively. The larger the area under the curve (AUC), the higher the prediction performance. We benchmarked our predictors on the independent test sets. We also compared the comprehensive predictor of RNAm5Cfinder with iRNA-PseColl and M5C-HPCR on the same independent test set. The binary (yes or no) prediction results of iRNA-PseColl and M5C-HPCR were obtained by submitting the RNA sequences to their servers.

### Construction of web-server platform

The user interface and message response mechanisms were based on JavaScript and Ajax. The data processing module was written by PHP5 and could process the input sequences into the numeric sequence encoding for subsequent random forest prediction.

## Conclusions

From above analyses, we can draw a conclusion that RNAm5Cfinder is an efficient tool to predict m5C sites. Comparing with other predictors, RNAm5Cfinder has two advantages: (1) Larger and more updated dataset, which together with the random forest machine learning framework, results in a better performance. (2) Ability to predict tissue-specific m5C sites. We believe that RNAm5Cfinder has great potentials and with more m5C site data become available, the performance of RNAm5Cfinder could be further improved.
